# What do readers need? Qualitative requirements of medical discharge summaries from the recipients’ perspective

**DOI:** 10.1186/s13690-025-01582-8

**Published:** 2025-04-14

**Authors:** Markus Unnewehr, Leonie Siemen, Hendrik Friederichs, Wolfram Windisch, Samer Zawy Alsofy, Bernhard Schaaf

**Affiliations:** 1 Department of Medicine, Faculty of Health, Witten/Herdecke University, Alfred- Herrhausen-Straße 50, 58448 Witten, Germany; 2 Department of Respiratory Medicine, Infectious Diseases, Sleep Medicine, Allergology, St. Barbara-Klinik, Am Heessener Wald 1, 59073 Hamm, Germany; 3 Faculty of Medicine, Lübeck University, Ratzeburger Allee 160, 23562 Lübeck, Germany; 4 Department of General and Visceral Surgery, Klinikum Dortmund, Beurhausstraße 40, 44137 Dortmund, Germany; 5 Medical Education Research Group, Medical School OWL, Bielefeld University, Bielefeld University, 33615 Morgenbreede, Bielefeld, Germany; 6 Department of Pneumology, Cologne Merheim Hospital, Kliniken der Stadt Köln gGmbH, Ostmerheimer Str. 200, 51109 Köln, Germany; 7 Department of Neurosurgery, St. Barbara-Klinik, Am Heessener Wald 1, 59073 Hamm, Germany; 8 Department of Respiratory Medicine, Infectious Diseases, Intensive Care Medicine, Klinikum Dortmund, Münsterstraße 240, 44145 Dortmund, Germany

**Keywords:** Discharge summary, Patient discharge, Healthcare communication, Transition of care, Patient safety, Population health

## Abstract

**Background:**

Discharge summaries (DSs) are the primary communication tools in clinical medicine. The transfer of information and plans is essential to ensure consistent patient safety and continuity of care. Therefore, DSs play a key role in population health. However, the overall quality of DSs is considered deficient, and there is a notable lack of scientific knowledge and research in this field, particularly regarding the needs of physicians as the primary recipients of DSs and key providers of ongoing patient care. The purpose of this study was to explore their requirements concerning the content, structure, and processing of DSs.

**Methods:**

A total of 159 outpatient primary care physicians (general practitioners, GPs) and specialists who refer patients to hospitals for various conditions were contacted across Germany using mixed sampling methods combining convenience, quota, and theory-driven sampling. Of these, 106 (66.67%) participated in telephone interviews. The interviews included nine open-ended questions, analyzed using Mayring’s qualitative content analysis, and a 43-item questionnaire, evaluated quantitatively with descriptive statistical methods to assess DS characteristics.

**Results:**

Quantitative analysis revealed that recipients rated the prompt arrival of DSs, a clear treatment and diagnostic plan, and a coherent rationale as the most important requirements. The least important elements were newsletter-style content, patient contact information, patient ethnicity, and hospital logos or awards. Both quantitative and qualitative analyses identified similar priorities and challenges in DS content and structure. Sending a diagnosis list was considered a top priority by all physicians. While GPs placed high importance on diagnoses, treatment plans, and medication changes, specialists prioritized a logical line of reasoning.

**Conclusion:**

This recipient-focused study highlighted specific areas for improvement in the content, structure, and delivery of DSs. Tailoring DS formats to the distinct needs of GPs and specialists has the potential to enhance their overall quality and utility. Ultimately, optimizing DSs may strengthen population health outcomes by improving care transitions, reducing adverse events, and supporting effective outpatient management across the healthcare system.

**Supplementary Information:**

The online version contains supplementary material available at 10.1186/s13690-025-01582-8.

**Table:** 

Text box 1. Contributions to the literature	
•DSs are the key communication tools in clinical medicine and therefore relevant elements of population health, but their quality and the understanding of recipients’ needs are lacking. •This study is the first comprehensive analysis of DS users’ perspectives. •Physicians appreciate diagnoses, timely delivery, treatment plans, and logical reasoning the most. In contrast, elements such as newsletters, patient contact details, ethnicity, consultation hours, study participation, logos and awards are the least important aspects. •The recipients' different priorities suggest that tailoring DS formats for GPs and specialists could improve DS quality.	

## Background

A discharge summary (DS) is a medical report that details a patient’s diagnosis, treatment, and follow-up care at the end of their hospital stay or outpatient specialty care. In Germany, it is the primary communication tool between healthcare providers, including hospital physicians, primary care physicians (general practitioners, GPs), and other treating physicians.

As a medical document, DSs are not primarily tailored to patients, who usually receive a copy of the DS from the issuing physician. In accordance with the principle of data sovereignty, allied health professionals such as physiotherapists, dietitians, nurses, or others may have access to relevant information with the patient’s consent. In other countries, depending on country-specific data protection requirements, care settings and institutional policies, this transfer of information is more automated and less dependent on individuals.

The primary purpose of a DS is to ensure continuity of care [1, 2] and to maintain patient safety [3–6]. A well-written DS is essential to ensure smooth transitions from hospital to outpatient care. When clearly structured and comprehensive, DSs provide healthcare providers with the information necessary to effectively continue treatment, reducing the likelihood of complications and avoidable hospital readmissions [3]. This is why DSs play a major role in population health.

Beyond individual patient care, optimizing DSs has broader implications for population health. Accurate discharge documentation simplifies early detection and management of chronic diseases, improves medication safety, and ensures that all patients receive clear and accessible health information, thereby reducing healthcare disparities. In addition, systematically collected discharge data contribute to epidemiological research by helping public health authorities to identify gaps in care and to develop targeted interventions to improve healthcare delivery.

The purpose of this study is to examine the perspectives of DS recipients, specifically GPs and outpatient specialists, regarding quality requirements. As the first point of contact for patients and the primary providers of ongoing out-of-hospital care, outpatient physicians are uniquely positioned to evaluate the content and structure needed to effectively support ongoing patient care and safety.

The results of this study are intended to serve as a foundation for improving the quality of DSs by establishing standards that enhance communication and promote patient safety.

While some English-speaking countries have implemented requirements for DSs to facilitate the effective transfer of information between healthcare professionals [7–9], similar guidelines or regulations are lacking in many other countries, including Germany. Furthermore, DSs have little place in medical school curricula and continuing medical education, although they are highly desired by young practitioners, especially those in primary care [10].

Given the significant volume of DSs generated daily, their production, processing, and review require substantial human and financial resources [11]. Despite this, formal education on DS quality, as well as persistent inadequacies in their preparation and handling, arelacking, which is difficult to understand. In addition, little effort has been made to address these deficiencies [12].

As a result, the quality of DSs is widely considered to be suboptimal. Common problems include missing relevant content despite established standards [9], failure to complete the summary on the day of discharge, and in some cases, failure to send it to the physician at all [13]. Such errors pose significant risks to patients [14].

While these errors may not directly impact financial reimbursement under the Diagnosis Related Group (DRG) system [15], they highlight the need for more accurate and comprehensive information, particularly in discharge medication orders [16, 17] and follow-up plans. These shortcomings pose serious risks to patient safety, particularly in the post-hospital care setting [18]. All of these deficiencies can lead to fragmentation of care and avoidable hospitalizations, which affect population health by increasing healthcare expenditures and consuming more medical resources than necessary.

Scientific knowledge about the perspectives and needs of DS recipients remains limited [10, 11, 19]. Existing standards and national guidelines are largely based on literature reviews and expert opinion, with some consideration of the perspectives of other stakeholders such as hospital administrators and health insurers [7–9, 20]. However, the lack of full involvement of these stakeholders, particularly primary care physicians, in the DS development process [13] highlights the suboptimal implementation of these standards.

Considering not only the content-related impact on population health, but also the large number of DSs generated daily with each patient discharge from a hospital or specialty care visit, these aspects underscore their fundamental role in the overall health care system.

## Methods

### Study design

We used a mixed-methods convergent parallel study design, incorporating qualitative and quantitative interviews to draw comprehensive conclusions [21, 22]. The study was conducted in collaboration with three major regional hospitals and three university hospitals in Germany.

An interview guide and a test instrument were developed, consisting of two parts: a questionnaire with nine open questions in the first part and 43 items in the second part, that participants were asked to rate. Because the item pool was intentionally large, the items were grouped thematically into six blocks (A–F; see Additional Supplement).

Standardized processes were implemented to minimize potential bias from confounders, effect modifiers, or selection bias. These processes included a study protocol, inclusion criteria, the establishment of subgroups, the interview guide and test instrument, the selection of the rating scale, transcription and coding rules, a rule-governed analysis, and a transparent report.

Ethical approval was obtained from the responsible regional ethical review board (Ethics Committee of the University of Lübeck, Germany, reference number 20–109). This approval encompassed adherence to all stipulated requirements for informed consent. The study was conducted without the involvement of any public, commercial, non-profit or other funding bodies or other external sponsors. There were no competing interests of the authors.

### Test instrument

To compile the questionnaire items, we analyzed the most relevant guidelines and similar studies prior to initiating the survey to identify key items [7–10, 19, 20]. The terms were then condensed using the Delphi method in two rounds, excluding items deemed self-evident or mandated by law.

For numeric values that could not be uniformly defined based on the literature, we deliberately did not restrict the items in the questionnaire. This approach aligned with the study’s objective to comprehensively explore the opinions of DS recipients. For example, it was not possible to extract an exact definition for the item “timeliness” from the literature, which provided varying definitions such as 24 hours [10], “within a reasonable timeframe” [9], at the time of discharge [20], or within one week [8].

### Participants’ characteristics

Participants were recruited through referrals from the involved hospitals as well as nationwide, utilizing a cluster and quota sampling approach [23] combined with convenience and theory-driven sampling methods [24]. This was done in a parallel multi-stage process. The participants were primarily contacted based on their specialty and the geographical area was extended where feasible. This ensured balanced group sizes between GPs and specialists. Convenience sampling was employed to augment the sample size, while theory-driven sampling was utilized to ensure the attainment of data saturation. This integrated approach combined the breadth of cluster sampling with the specificity of quota sampling. Sampling ensured balanced representation regarding outpatient practice type and medical specialty [23]. One group of participants consisted of GPs, while the other consisted of specialists. The inclusion criteria required participants to be DS recipients, while the exclusion criteria included doctors who did not receive DSs and those who declined to participate in the interviews.

Data saturation and sample size planning posed challenges in this study due to the mixed-methods approach. Limitations, such as a fixed time frame to capture a realistic representation of participants’ situations under consistent conditions and the large number of items, added to the complexity. After careful consideration - including the impracticality of conducting meaningful t-tests due to the large number of different items - a sample size of at least 100 was determined to achieve a Gaussian distribution [25]. This size was also chosen in order to ensure simplicity, universality, and comparability of the quantitative results as well as a clearer presentation of the findings, while the sample sizes in the three subgroups are adequate and representative. Data saturation for the qualitative part was continuously monitored during the interviews. The final sample size met all the criteria outlined.

### Interviews

Interviews were conducted following COREQ (Consolidated Criteria for Reporting Qualitative Research [26]). In addition, other quality assurance measures were implemented [25]. Personal perspectives and previous experiences of the researchers might have influenced the data collection and interpretation process. The relationship between the interviewer (L. S.) and participants was strictly professional, with no prior personal relationships, ensuring unbiased interactions, and focused solely on the research context. To reduce potential bias, standardized interview protocols and structured scripts were strictly followed. Regular peer debriefing sessions in the research group and continuous self-reflection were implemented to critically assess and address emerging assumptions. Furthermore, all interviews were pseudonymized and subjected to iterative coding by the interviewer and checked by the other researchers, ensuring a balanced and transparent analysis.

Telephone interviews were conducted by L.S. from her home between March 1 and October 31, 2018, using a standardized script (see Additional Supplement). A pilot test of the instrument had previously been conducted with eleven colleagues and medical students to ensure accuracy. Telephone contact was made directly or through the practice staff. Audio recordings were made without accompanying field notes. The interview guide and the test instrument were presented after the study and its purpose had been explained. The duration of the interviews ranged from 6 to 32 minutes, with no repeat interviews or follow-ups. No feedback on the results was obtained from the interviewees. The files were pseudonymized during transcription, which was done according to defined rules and then cross-checked. The audio files and participants’ names were deleted. Participants did not receive a transcript.

### Data analysis

Following the COREQ standard and checklist [26], content analysis was applied to the large amount of written data obtained after pseudonymized transcription.

Mayring’s qualitative content analysis was chosen because it is particularly suitable for evaluating large data sets. This approach is a systematic, rule-guided method designed to manage large amounts of textual data, such as interview transcripts. Unlike open-ended approaches such as thematic analysis, Mayring’s method integrates both deductive and inductive coding, allowing predefined categories to be refined iteratively in light of the data. This structured process enhances transparency and reproducibility by applying explicit coding rules. In contrast to grounded theory, which emphasizes the development of theory from the data, Mayring’s approach is particularly suited to producing a consistent and verifiable summary of content. While elements of grounded theory were incorporated into the inductive revision of our category system, other methodological orientations — such as discourse analysis, ethnography, or phenomenology — were deemed less appropriate for addressing our research questions.

The study results were categorized using a combination of structuring and summarizing content analysis. The nine open-ended questions served as deductive categories, which guided the structuring of the text corpus and led to the development of a preliminary category system. The rule-guided process involved multiple iterations of the transcripts, including checks of the coding guide and the initial category system. Revisions were performed inductively, incorporating aspects of grounded theory analysis into the content analysis. As a result, the qualitative findings were presented in a tabular final category system consisting of eleven categories with subcategories, including major and minor themes (Additional Supplement) [27].

Quantitative data were analyzed using IBM SPSS™ Statistics (Version 29, Armonk, NY, USA) and Microsoft Excel™ 2021 (Redmond, WA, USA). Descriptive statistics such as median, mean, range, interquartile range (IQR), empirical variance, and standard deviation (SD) were calculated. Normality of the data distribution was assessed using the Shapiro-Wilk and Kolmogorov-Smirnov tests. Bivariate analysis was performed using Pearson correlation with two-tailed significance, excluding the coefficient of determination. A strong correlation was defined as a correlation coefficient >|0.5| with significance at the 0.01 level (two-sided).

The study also examined the effects of confounders, including medical specialty designations.

## Results

A total of 159 outpatient physicians were contacted, and 106 of them participated in the survey, yielding a response rate of 66.67%. There were no dropouts.

Of the interviewees, 31 were female (29.2%). The mean age of participants was 50.07 years (median 49.5, SD 9.41, IQR 12.8), with an age range from 30 to 82 years. Female participants had a mean age of 46.48 years (median 46, SD 6.67, IQR 10.5), while male participants 51.55 years (median 52, SD 10, IQR 12.5).

Of the 106 participants, 48 were GPs, and 58 were specialists. 53 participants had been practicing outpatient care for less than nine years.

### Qualitative part

According to the interviewees (Table 1), DSs are an essential means of communication among healthcare practitioners. General content requirements include completeness and thoroughness in addressing admission questions.

**Table 1:** Findings from Mayring’s qualitative content analysis of interviews with 106 outpatient physicians in Germany (March–October 2018)– study on qualitative requirements for medical discharge summaries from the recipients’ perspective

**A. Language used in discharge summaries**	1. Essential and precise content without general empty phrases
	2. Correct application of medical terms without abbreviations
	3. Professional language understandable across departments
	4. Use of short, precise sentences and correct spelling and grammar, although errors can be tolerated
**B. Outline in terms of content**	1. Clear structure („continuous thread“)
	2. Visibility of essential information at first glance
	3. Consistency of content between the individual sections
	4. Emphasis on content-bearing sections
**C. Formatting and layout**	1. Clarity by inserting paragraphs
	2. Limitating length without omitting information
	3. Use of legible writing
	4. Highlighting important information or sections
	5. Readability even after multiple scans/copies
	6. Limited space for logos
**D. Organizational considerations**	1. Timely arrival and reliable delivery of discharge summaries
	2. Review of medical reports by medical specialists or senior physicians
**E. Purpose of discharge summaries**	1. Communication tool for information sharing between all practitioners
	2. Legal protection for the author
	3. Presentation of a medical case and hospital course
	4. Recommendations and explanations of further treatment and/or diagnostics
**F. General content requirements**	1. Completeness for effective information transfer
	2. Clear explanation of admission reason and outcomes
**G. Diagnoses**	1. Correct and complete diagnoses in a structured and clear presentation
	2. Definite, precise description of the disease, especially regarding the current hospital stay
	3. Supplementing the diagnoses with sub-items
**H. ** **Medical summary and assessment**	1. Essential part of discharge summaries
	2. Complete summary and assessment of an individual medical case
	3. Structured presentation of a patient case with a focus on substantial information
**I. Medication**	1. Correct, complete medication plan consistent with pre-hospital medication
	2. Clearly marked changes to a patient’s medication regimen
**J. Examinations carried out and findings**	1. Examination findings, e.g. as an appendix
	2. Important results only, exclude normal findings or repeats
	3. Limit the length of the result texts; focus on result/evaluation
	4. Relevant and/or pathological laboratory values
**K. Therapy und procedures /** **therapy recommendation**	1. Concrete recommendations for post-discharge therapy
	2. Summary of hospital therapies and procedures
**L. Medical history / physical examination**	1. Complete medical history and physical examination findings at the time of admission

The participants emphasized the importance of concise expression, advocating for short sentences, correct language, and precise terminology. Text modules and abbreviations were generally discouraged by the participants. A logical and structured presentation of facts, with important information such as diagnoses and medical history highlighted in a well-organized format, was preferred. Clear treatment recommendations, including follow-up appointments and a detailed medication plan, were also highly valued. Additionally, DSs should include concise descriptions of examinations, procedures, and findings.

Criticism was raised regarding the lack of logical connections within DS sections (e.g., diagnoses not aligning with prescribed medication). Participants highlighted the need for clarity in form and layout, including the use of paragraphs, brevity, legible fonts, and the avoidance of handwritten reports, colored paper, and excessive use of logos. Timely and reliable delivery of DSs and accurate recipient addresses were considered important.

Examples of representative quotes from participants can be found in the Additional Supplement.

### Quantitative part

**Fig. 1:**
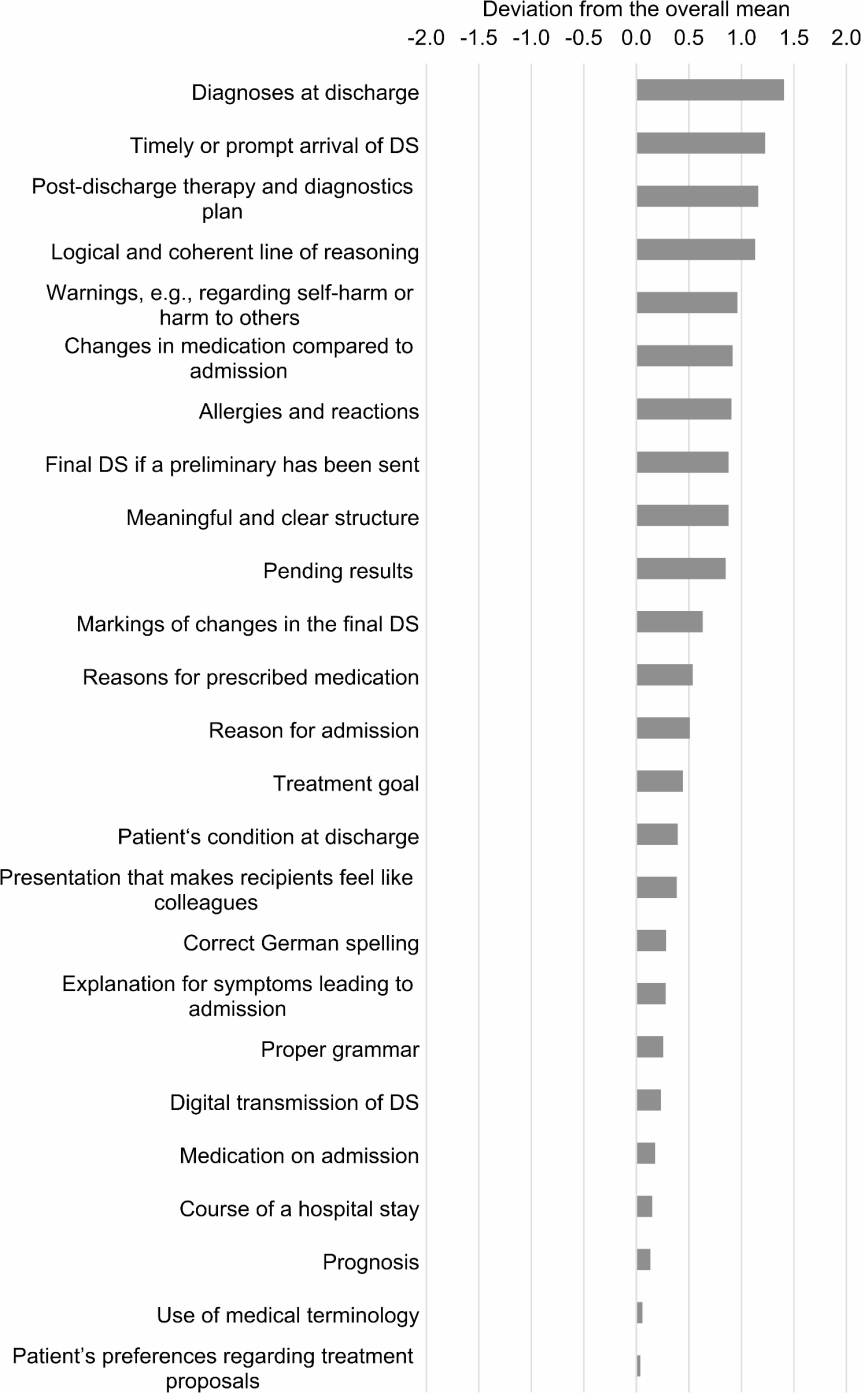
Positive deviations of item means from the overall mean of 3.36, calculated as the arithmetic mean of all item means, ordered from highest to lowest deviation. Analysis of interviews with 106 outpatient physicians in Germany (March–October 2018) on qualitative requirements for medical discharge summaries from the recipients’ perspective

**Fig. 2:**
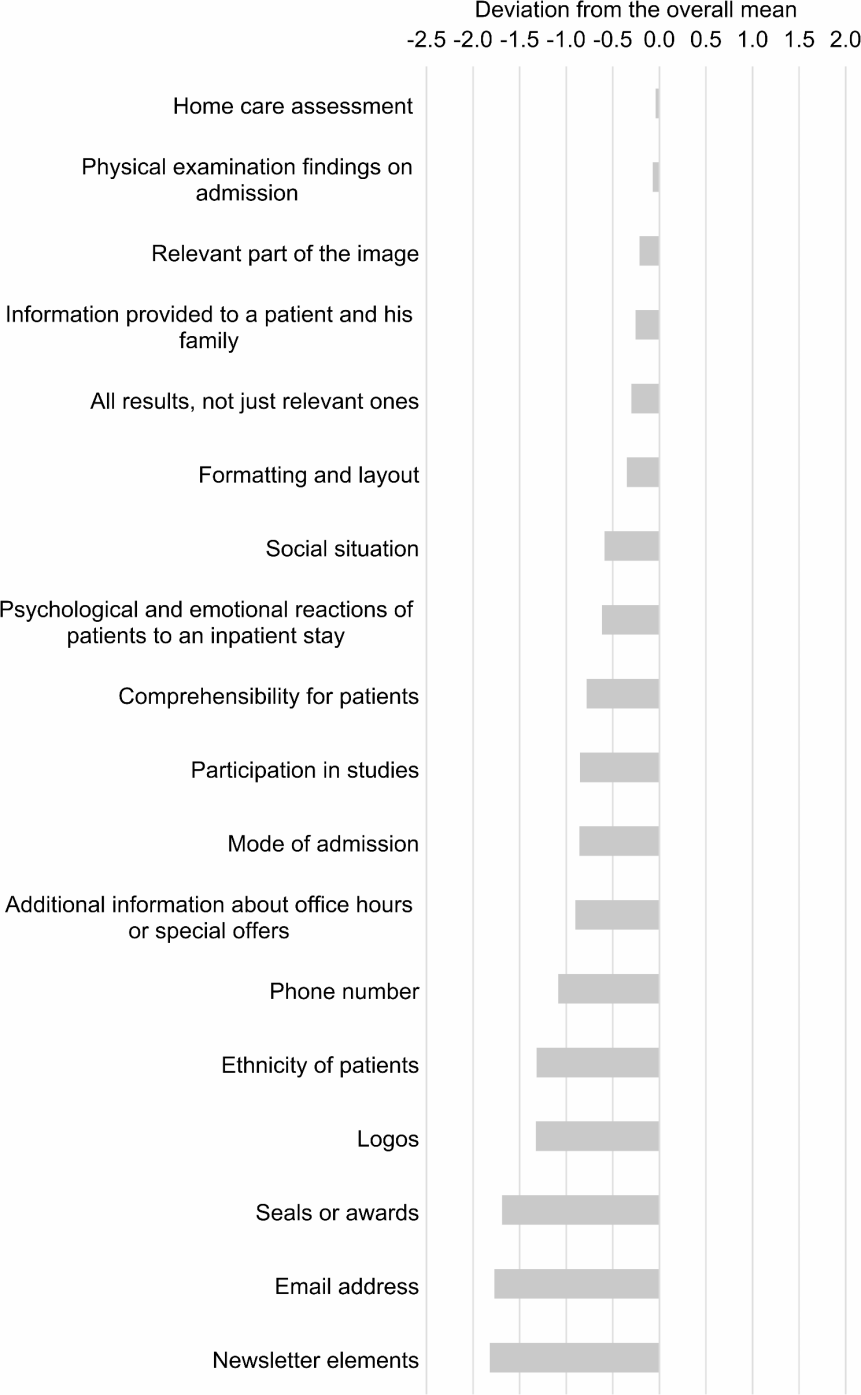
Negative deviations of item means from the overall mean of 3.36, calculated as the arithmetic mean of all item means, ordered from highest to lowest deviation. Analysis of interviews with 106 outpatient physicians in Germany (March–October 2018) on qualitative requirements for medical discharge summaries from the recipients’ perspective

To support the optimization of DSs, a ranking of participants’ evaluations was derived (Table 2). Figures 1 and 2 illustrate the weighting of items, based on the deviations of their individual means from the overall average mean of all means of the items (3.36, corresponding median 3.51), ordered by deviation from the overall mean, from highest to lowest. These figures highlight items that are more desired (Fig. 1) and less desired (Fig. 2), using the overall average mean as a reference line.

**Table 2:** Ranking of questionnaire items by mean values in the comparison between GPs and specialists (*n* = 106 outpatient physicians, Germany, March–October 2018)– study on qualitative requirements for medical discharge summaries from the recipients’ perspective. The overall mean of 3.36 was calculated as the arithmetic mean of all item means

Item	All physicians	GPs	Specialists
	Means		
Diagnoses at discharge	4.76	4.79	4.74
Timely or prompt arrival of DS	4.58	4.50	4.66
Post-discharge therapy and diagnostics plan	4.52	4.63	4.43
Logical and coherent line of reasoning	4.49	4.29	4.66
Warnings, e.g., regarding self-harm or harm to others	4.32	4.27	4.36
Changes in medication compared to admission	4.27	4.44	4.14
Allergies and reactions	4.26	4.38	4.17
Final DS if a preliminary has been sent	4.24	4.21	4.26
Meaningful and clear structure	4.24	4.15	4.31
Pending results	4.21	4.27	4.16
Markings of changes in the final DS	3.99	4.23	3.79
Reasons for prescribed medication	3.90	3.98	3.83
Reason for admission	3.87	3.77	3.95
Treatment goal	3.80	3.75	3.84
Patient‘s condition at discharge	3.75	3.81	3.71
Presentation that makes recipients feel like colleagues	3.74	3.43	4.00
Correct German spelling	3.64	3.35	3.88
Explanation for symptoms leading to admission	3.64	3.48	3.77
Proper grammar	3.61	3.35	3.83
Digital transmission of DS	3.59	3.51	3.65
Medication on admission	3.54	3.45	3.61
Course of a hospital stay	3.51	3.60	3.43
Prognosis	3.49	3.43	3.53
Use of medical terminology	3.42	3.20	3.60
Patient’s preferences regarding treatment proposals	3.40	3.35	3.43
Home care assessment	3.32	3.60	3.07
Physical examination findings on admission	3.29	3.21	3.34
Relevant part of the image	3.14	2.64	3.56
Information provided to a patient and his family	3.10	3.15	3.07
All results, not just relevant ones	3.06	3.15	2.98
Formatting and layout	3.01	2.71	3.24
Social situation	2.77	2.81	2.73
Psychological and emotional reactions of patients to an inpatient stay	2.74	3.04	2.50
Comprehensibility for patients	2.58	2.61	2.55
Participation in studies	2.51	2.10	2.80
Mode of admission	2.50	2.40	2.58
Additional information about office hours or special offers	2.46	2.67	2.29
Phone number	2.27	2.50	2.10
Ethnicity of patients	2.04	1.83	2.20
Logos	2.03	1.80	2.20
Seals or awards	1.67	1.71	1.64
Email address	1.59	1.50	1.64
Newsletter elements	1.54	1.79	1.34
**Overall mean**	**3.36**	**3.32**	**3.39**

Across the specialties, interviewees agreed that diagnoses are an essential component of DSs. Ten items received mean values greater than 4 from all participants, classifying them as very important according to the scale used (Table 2). These included, for example, the timely arrival of the DS, precise therapy guidance, and a clear diagnostic plan. A DS should follow a logical structure and present information coherently. Key information such as warnings, medication changes, allergy details, and preliminary or pending results must not be omitted.

The thematic blocks A–F, shown in the tables and figures in the Additional Supplement, provide further insights. The analysis indicated that all items within Block B were considered at least “important” (mean > 3) according to the scale, underscoring their essential nature. “Diagnoses at discharge” had the highest mean, with the lowest standard deviation (SD) and interquartile range (IQR), indicating strong agreement among participants. In Block C, the low SD reflected a shared perspective on aspects such as “post-discharge therapy and diagnostic plan,” which were among the most highly rated items. The results from Block D (External Impact of a DS) suggested that the DS’s institutional origin plays a less significant role. Blocks A (Admission and general data) and E (Structure and linguistic form) included items with divergent importance ratings.

Aspects such as newsletter elements, seals, logos, and non-medically relevant information (e.g., email addresses, phone numbers, comprehensibility for patients, social situation; Table 2) were rated as having low importance.

The subgroup comparison (Additional Supplement) revealed that GPs prioritized medical aspects such as treatment recommendations, follow-up plans, diagnostic details, medication changes, allergy information, and clear identification of new or updated content within the DS. They also sought information on the patient’s social context and overall medical status. Specialists, by contrast, placed more emphasis on the appearance and structure of the DS, as well as the use of precise medical language, favoring a logical and meaningful presentation.

Five pairs of items showed a strong correlation, as defined above:

Course of a hospital stay (item No. B2_04) and explanation for symptoms leading to admission (item No. B2_05), correlation coefficient (r) = 0.561.

Reasons for prescribed medication (B2_06) and changes in medication compared to admission (B2_07), r = 0.512.

Correct German spelling (B5_02) and proper grammar (B5_03), r = 0.901.

Relevant part of the image (B4_06) and formatting and layout (B6_08), r = 0.520.

Timely or prompt arrival of DS (B6_06) and warnings, e.g., regarding self-harm or harm to others (B6_07), r = 0.512.

Further details are presented in the tables and figures in the Additional Supplement.

These correlations highlight the close connection between content clarity, linguistic accuracy, and timely delivery. They underscore the need for a cohesive approach to DS quality that integrates both structural and informational precision.

### Integration of quantitative and qualitative data

The combined analysis of qualitative and quantitative findings highlights key areas of consensus among participants. Both data sets demonstrated a shared preference for DSs prioritizing essential medical content, including diagnoses, medication details, follow-up plans, and relevant findings, alongside timely transmission to recipients. Quantitative analysis reinforced the importance of items such as clear therapy guidance, structured content, and warnings (e.g., about risks to the patient or others), which aligned with qualitative feedback emphasizing concise, accurate information and logical organization while avoiding vague or non-essential details.

The subgroup analysis revealed notable differences in emphasis: GPs prioritized post-discharge therapy plans and the clear identification of new or allergy-related information, while specialists valued logical and meaningful structure and prompt receipt of the DS. Both groups ranked non-medical elements, such as logos and unnecessary personal details, as low priorities. This shared preference for clinically relevant, streamlined content underscores the need for DS improvements focused on timely, structured delivery and information that directly supports the continuity of patient care. Such improvements reflect not only individual care needs but also the broader imperative to enhance healthcare coordination and achieve better population-level outcomes through standardized communication.

## Discussion

To the best of our knowledge, this study is the first in the literature to comprehensively analyze DS recipients’ requirements across a broad range of elements, incorporating perspectives from both GPs and specialists. The qualitative approach in our mixed-methods design enabled an open and in-depth exploration of the main stakeholders’ perspectives, while the quantitative component complemented these insights with structured, comparable data.

The qualitative results revealed a heterogeneous picture of DS requirements, reflecting the spontaneous and unrestricted reactions of participants. This variability underscores the authenticity of the findings.

The composition of the sample included 48 GPs (45.28%) and 56 specialists, equally divided between respiratory physicians (27.36%) and other specialists (27.36%). This distribution was not considered a significant confounder, as GPs, the primary recipients of DSs, were the majority, and the results within the specialist group were consistent.

Direct phone interviews yielded more authentic responses than written questionnaires [28], which might have resulted in minimal responses due to the extensive number of items [19, 29]. Although conducted in 2018, data analysis was delayed by the COVID-19 pandemic. Literature review and observations suggest that DS management practices remained largely unchanged during this period [30], preserving the relevance of the findings. This assumption is supported by the absence of major policy reforms or guideline changes related to DS preparation or transmission in Germany between 2018 and 2024, as confirmed by national healthcare reports and the unchanged content of relevant medical education curricula during this period.

The design of a DS has traditionally been based on established patterns [3, 31, 32], though few elements are universally recognized as “clear” or “self-evident.” Timely transfer of information remains a critical requirement, as delays can impede subsequent patient care [5].

The preferred DS structure identified in this study aligns with traditional models but includes refinements suggested by participants, such as rapid production, avoidance of handwritten content, precise language, and clear layout using organized paragraphs and strategic highlighting.

The discrepancies in DS requirements across medical specialties identified in our study align with daily clinical practice, supporting the decision to group interviewees by specialty.

Subgroup analysis revealed that GPs prioritized content completeness and clarity of the DS. They emphasized the importance of understanding patients’ informed status and current situation, as they are the primary medical contact for patients. GPs also expect post-discharge treatment and diagnostic plans, as well as updates on medication changes (Table 2), reflecting their role in ensuring continuity of care.

In contrast, specialists placed greater emphasis on the logical and clear structure of the DS, focusing on presentation aspects such as the admission mode and discharge condition. They considered non-specialty diagnoses less relevant, as treatment decisions beyond their expertise were not their responsibility.

This recipient-focused study aligns with recent studies on DS structure and content, supporting our findings. For example, Unnewehr et al.‘s systematic review emphasized transparency and consistency for recipient satisfaction [3], findings corroborated by our empirical data.

Our test instrument was informed by expert opinions, authoritative documents, public input, patient experiences, and literature reviews [7–9, 20], consistent with the approach of other empirical studies [1, 30, 32, 33].

For instance, Belleli et al.‘s audit showed timely DS receipt but frequent omission of key information like medication changes and referrals [1], deficits also noted in our study. In contrast to Belleli’s focus on content availability, our work highlights perceived content relevance from the recipient’s perspective.

Chatterton et al. found GPs irritated by overly long, irrelevant DSs [32]. Our participants preferred clear action items and follow-up notes, which we quantified through our assessment tool. Similarly, Rash et al. found that brevity, structure, and interpretation were preferred over raw data [30], aligning with our finding that interpretive, forward-looking content (e.g., follow-up plan) was rated higher.

Silver et al.’s national survey in the United States ranked hospital course, diagnoses, medication reconciliation, and follow-up as DS priorities [33], reflecting our interviewees’ preferences.

International studies validate our findings [10, 19]. Yemm et al. found UK GPs valued medication changes more than junior doctors addressed them [10], echoing our observation of varied recipient expectations. Mahfouz et al. tested a DS assessment tool aligned with GP priorities in Australia, further supporting our instrument.

Bachmann et al. reported German GPs received unclear DSs with poor structure and excessive abbreviations [11]. While focusing on language clarity, their findings align with our detailed content-based evaluation.

Wimsett et al. identified discharge diagnosis, treatment, investigation results, and follow-up as the most critical DS elements in a systematic review, aligned with the critical domains of our evaluation tool [29].

Bansard et al. used a Delphi process to develop standardized DS templates in France with various stakeholders like GPs and patients [34]. Their emphasis on standardized forms and prompt production supports our conclusion that recipient feedback should guide DS improvement efforts.

Recurring deficits in DSs despite existing standards [13, 32, 35] pose patient safety risks [14]. Al-Damluji et al. found inconsistent DS quality and timeliness across hospitals [13], while Mishra et al. highlighted missing elements such as discharge status and physician identification [35], also observed in our study.

Williams et al. found over half of DS communication errors caused harm, especially in medication and follow-up, which were top concerns for our recipients [14].

Training in DS writing during medical education and residency is essential [28, 36–39]. While quality improvement projects (QIPs) have improved DSs through structured feedback [36, 40–42], many lack sustainability. Myers described improved DS quality through structured curricula and feedback [40]. Fasal et al. demonstrated gains in documentation through workshops and checklists [36]. Scarfield et al. achieved compliance with DS standards through stakeholder feedback and plan-do-study-act (PDSA) cycles [37]. Patel et al. reported increased DS quality and GP satisfaction through template redesigns and audits [28]. Shaikh et al. improved DS completion and content in a pediatric setting [38], and Legault et al. found frequent inaccuracies and follow-up gaps in DSs from junior doctors [39].

In total, these studies support the conclusion that training alone, while beneficial, is insufficient. Our results indicate that structured templates, assessment tools, and feedback tailored to recipients’ needs are required. The recipient requirements identified here reinforce the need to embed DS education in curricula, supported by empirical evaluation and continuous quality assurance. Focusing on the most relevant items (Table 2) may improve DS quality and streamline communication.

With a sample size of 106, normal distribution can be assumed [43], as confirmed by testing. Descriptive analyses fulfilled the study’s aim to objectively map DS recipients’ needs. This sets a foundation for future research into DS structure, timing, and specialty-specific standards.

High-quality DSs are crucial for smooth care transitions. Beyond individual care, optimized DSs impact population health by supporting chronic disease management, enhancing safety, and ensuring accessible health information. They help reduce disparities, prevent readmissions, and enable public health efforts to identify care gaps and design targeted interventions.

Our findings suggest that recipient-tailored DS formats improve patient care, enhance system efficiency, reduce health disparities, and support effective public health interventions. Embedding public priorities into DS design can contribute to a more resilient healthcare infrastructure.

## Conclusion

This study highlights the critical role of DSs in medical communication and identifies deficiencies from the recipients’ perspective.

Important requirements (e.g., diagnoses, timely delivery, treatment and diagnostic plans, logical reasoning) and less relevant elements (e.g., newsletter elements, patient contact details, ethnicity, consultation hours, participation in studies, logos, and awards) were identified. Differences between the views of GPs and specialists were also observed.

Future research should focus on specific aspects of DSs, such as the diagnosis list, including the assessment current DS quality, the development of practical solutions for simplifying DS processing, and the evaluation of the impact of DSs on individual patient care and population health outcomes.

Optimizing DS quality supports individual care and strengthens population health by reducing care fragmentation, preventing avoidable events, and enabling effective epidemiological surveillance. Moreover, high-quality DSs can serve as a cornerstone for standardized data collection, supporting real-time monitoring, resource planning, and health policy development.

Quantifying the impact of DS improvements on patient outcomes and healthcare system efficiency remains a key research priority. These efforts are essential to improve health at both the patient and population levels and to strengthen healthcare systems amid rising rates of chronic disease.

## Electronic supplementary material

Below is the link to the electronic supplementary material.

Supplementary Material 1

## Data Availability

No datasets were generated or analysed during the current study.

## Abbreviations

COREQ: Consolidated criteria for reporting qualitative research.
DS: Discharge summary
GP: General practitioner
PDSA: Plan-do-study-act
QIP: Quality improvement project
