# Supplementary material for: What do readers need? Qualitative requirements of medical discharge summaries from the recipients’ perspective
Source: Arch Public Health. 2025 Apr 14;83:104. doi: 10.1186/s13690-025-01582-8 (PMC11995522; doi:10.1186/s13690-025-01582-8)
Supplement: Supplementary file 1 — Supplementary Material 1 [file 13690_2025_1582_MOESM1_ESM.docx]

Unnewehr M et al.

**What do Readers need? Qualitative Requirements of Medical Discharge Summaries from the Recipients’ Perspective**

**Additional Supplement**

Methods

Original Interview Script in English language

Relevant quotes

Tables 1-11 and Figures 1-12

**Methods**

The qualitative part included questions concerning the content, purpose, language, and format of the report. Participants were asked to answer these questions freely at their own discretion. For this reason, the interview was started with this open approach, followed by the evaluation part.

Due to the intentionally large scope of the 43 items, they were grouped thematically:

A Admission and General Data

B Results and Course of Treatment

C Post-Hospitalization Procedures

D External Impact of DS

E Structure and Linguistic Form

F Miscellaneous

After general open questions on their view to DS, participants were asked to rate each item on a Likert scale from 1 to 5, according to their opinion of the importance of the item in DS, with 1 = barely important, 2 = mildly important, 3 = important, 4 = very important, 5 = extremely important. Items not applying to the participant could be skipped.

## Original Interview Script in English language

Interview guide

1. Greeting and introduction of the interviewer on the phone

- Call in cooperation with the hospital and the university

2. Presentation of key information on the research project and the survey conducted, including its methodology

- Survey on the topic of “Quality of discharge summaries” on the phone

- Objective: To identify the requirements of discharge summaries from the point of view of recipients, to identify differences in quality

- Duration of around ten minutes

- Offer to make an appointment for the interview, if desired

- Every time during the interview: room for questions from the participant

3. Explanation of the data protection measures and obtaining verbal consent before the interview

- Verbal explanation of the study intention and data protection measures

- Obtaining the study participants' consent to the audio recording of the entire interview

- Consent to data processing for the purpose of the study and to data archiving until the recorded interview is written down with consecutive pseudonymization and deletion

- Exception: certain personal data (see point 4.), which may only be used in pseudonymized form within the framework of the present study, even after transcription, with the consent of the interviewees

4. Demographic data of the participants

- Period of time of outpatient medical practice, acquired specialty designations and demographic data (age, gender)

5. concrete explanation of the survey procedure and content

- Two parts of the survey: narrative (qualitative) and targeted evaluation (quantitative) part

- Explanation of the procedures before the start of each part

6. Narrative question part

- Request to answer the questions as spontaneously and honestly as possible

- Reading out the nine open questions from the test instrument

- Possible discussion impulses in case of missing answers/message not delivered:

o “In your experience, what is more often ignored or inadequately addressed by discharge summaries’ authors in this regard?”

o “You have a lot of experience with discharge summaries. What do authors of often do wrong here?”

7. Targeted evaluation section

- Explaining the plan to now read out a list of possible requirements for discharge summaries

- Asking to rate each of these using a defined scale

- Exact explanation of the scale:

o 5: extremely important

o 4: very important

o 3: important

o 2: less important

o 1: hardly important

- request to make full use of the scale

- Note: If a point does not seem relevant for one, asking to formulate this as follows: “Not relevant for me”, and in this case not giving a rating

8. Final questions, thanking for the participation

- Renewed request as to whether the data collected may be evaluated and used in an anonymized/pseudonymized form as part of the present study on the quality of discharge summaries

- “Would you be willing to participate in a quality assessment of discharge summaries in the future?”

- Discussion of any unanswered questions

- Thanking for the participation

**Relevant quotes**

A. "There are, of course, medical terms in it, that's clear, but increasingly in the inpatient sector, we miss that colleagues are not able to explain it in appropriate German." (Source: H_B_001; F7); "for ticking like Multiple Choice" (Source: P_G_002; F2); "no nested sentences" (Source: N_S_004; F6)

B. "Clear structure, diagnoses, brief course of the patient’s stay in the hospital, and the therapy recommendation also clearly structured: How do we continue? What's next?" (Source: H_L_001; F1)

C. "Clear! Yes, it doesn't have to be fancy and certified according to this and that, golden prize coin here and there, and what else you can imagine, what is sometimes there to embellish." (Source: H_B_001; F9)

D. "quickly delivery" (Source: P_H_003; F1); "pending results are not reported" (Source: P_H_003; F2); "What we often notice is that these medical reports apparently are no longer controlled, no longer read, but they are dictated and then simply sent." (Source: H_B_001; F2)

E. "Ultimately, the purpose is, of course, after the inpatient stay, a complication-free transition to the outpatient sector and continuation of the initiated measures." (Source: H_B_001; F3)

F. ... ("And then there are good medical reports that contain the latest diagnoses [...]" (Source: H_B_001; F8))

G. "the correct diagnosis in the order of importance of the diagnoses." (H_J_001; F8)

H. "... there is often also a lack of discussion, considerations, why a certain disease is considered likely and why other diseases or causes are excluded." (Source: H_M_001; F2)

I. "For example, just five minutes ago, I received a medical report, and it says at the front: allergy to metamizol, and in the medication list, it says: metamizol 4x2 tablets. Now, of course, I don't know: does she have an allergy? Does she not? Did she take it or did she not take it?" (Source: H_B_001; F2)

J. "[...] Five times the same lab results, that's annoying [...]" and "[...] very often, I notice that lab tests, for example, are attached three or four times [...]" (Source: H_G_006; F1 and F2)

K. "A detailed description of the medical history, i.e., the anamnesis" (Source: H_M_001; F1)

Tables and Figures

| **Item** | **n total** | **n missing** | **Mean** | **Median** | **SD** | **Variance** | **Interquartile Range** | **Range** | **Minimum** | **Maximum** |
| --- | --- | --- | --- | --- | --- | --- | --- | --- | --- | --- |
| Reason for admission | 106 | 0 | 3,87 | 4,00 | 0,85 | 0,72 | 1,75 | 3 | 2 | 5 |
| Medication on admission | 104 | 2 | 3,54 | 3,50 | 1,07 | 1,14 | 1 | 4 | 1 | 5 |
| Physical examination findings on admission | 105 | 1 | 3,29 | 3,00 | 1,06 | 1,12 | 1 | 4 | 1 | 5 |
| Social situation | 104 | 2 | 2,77 | 3,00 | 0,96 | 0,93 | 1 | 4 | 1 | 5 |
| Participation in studies | 93 | 13 | 2,51 | 2,00 | 1,31 | 1,73 | 3 | 4 | 1 | 5 |
| Mode of admission | 104 | 2 | 2,50 | 3,00 | 1,06 | 1,12 | 1 | 4 | 1 | 5 |
| Phone number | 88 | 18 | 2,27 | 2,00 | 1,36 | 1,85 | 2 | 4 | 1 | 5 |
| Ethnicity of patients | 98 | 8 | 2,04 | 2,00 | 0,86 | 0,74 | 1 | 3 | 1 | 4 |
| Email address | 75 | 31 | 1,59 | 1,00 | 0,93 | 0,86 | 1 | 4 | 1 | 5 |

**Table 1: Results block A Admission and General Data. Analysis of interviews with 106 outpatient physicians in Germany (March–October 2018) on qualitative requirements for medical discharge summaries from the recipients’ perspective.**

**Figure 1: Rating distribution block A Admission and General Data. Analysis of interviews with 106 outpatient physicians in Germany (March–October 2018) on qualitative requirements for medical discharge summaries from the recipients’ perspective.**

| **Item** | **n total** | **n missing** | **Mean** | **Median** | **SD** | **Variance** | **Interquartile Range** | **Range** | **Minimum** | **Maximum** |
| --- | --- | --- | --- | --- | --- | --- | --- | --- | --- | --- |
| Diagnoses at discharge | 106 | 0 | 4,76 | 5,00 | 0,61 | 0,37 | 0 | 4 | 1 | 5 |
| Changes in medication compared to admission | 106 | 0 | 4,27 | 4,00 | 0,83 | 0,69 | 1 | 3 | 2 | 5 |
| Allergies and reactions | 106 | 0 | 4,26 | 5,00 | 0,88 | 0,78 | 1 | 3 | 2 | 5 |
| Pending results | 105 | 1 | 4,21 | 4,00 | 0,85 | 0,72 | 1 | 3 | 2 | 5 |
| Reasons for prescribed medication | 106 | 0 | 3,90 | 4,00 | 1,00 | 1,00 | 2 | 4 | 1 | 5 |
| Patient's condition at discharge | 105 | 1 | 3,75 | 4,00 | 0,92 | 0,86 | 2 | 3 | 2 | 5 |
| Explanation for the symptoms leading to admission | 105 | 1 | 3,64 | 4,00 | 1,00 | 1,00 | 1 | 4 | 1 | 5 |
| Course of a hospital stay | 106 | 0 | 3,51 | 3,00 | 1,01 | 1,03 | 1 | 4 | 1 | 5 |
| All results, not just relevant ones | 106 | 0 | 3,06 | 3,00 | 1,16 | 1,34 | 2 | 4 | 1 | 5 |

**Table 2: Results block B Results and Course of Treatment. Analysis of interviews with 106 outpatient physicians in Germany (March–October 2018) on qualitative requirements for medical discharge summaries from the recipients’ perspective.**

**Figure 2: Rating distribution block B Results and Course of Treatment. Analysis of interviews with 106 outpatient physicians in Germany (March–October 2018) on qualitative requirements for medical discharge summaries from the recipients’ perspective.**

| **Item** | **n total** | **n missing** | **Mean** | **Median** | **SD** | **Variance** | **Interquartile Range** | **Range** | **Minimum** | **Maximum** |
| --- | --- | --- | --- | --- | --- | --- | --- | --- | --- | --- |
| Post-discharge therapy and diagnostics plan | 106 | 0 | 4,52 | 5,00 | 0,66 | 0,44 | 1 | 2 | 3 | 5 |
| Treatment goal | 106 | 0 | 3,80 | 4,00 | 0,86 | 0,75 | 1 | 3 | 2 | 5 |
| Prognosis | 104 | 2 | 3,49 | 3,00 | 0,85 | 0,73 | 1 | 4 | 1 | 5 |
| Home care assessment | 101 | 5 | 3,32 | 3,00 | 0,92 | 0,85 | 1 | 4 | 1 | 5 |

**Table 3: Results block C Post-Hospitalization Procedures. Analysis of interviews with 106 outpatient physicians in Germany (March–October 2018) on qualitative requirements for medical discharge summaries from the recipients’ perspective.**

**Figure 3: Rating distribution block C Post-Hospitalization Procedures. Analysis of interviews with 106 outpatient physicians in Germany (March–October 2018) on qualitative requirements for medical discharge summaries from the recipients’ perspective.**

| **Item** | **n total** | **n missing** | **Mean** | **Median** | **SD** | **Variance** | **Interquartile Range** | **Range** | **Minimum** | **Maximum** |
| --- | --- | --- | --- | --- | --- | --- | --- | --- | --- | --- |
| Digital transmission of DS | 98 | 8 | 3,59 | 4,00 | 1,16 | 1,35 | 1 | 4 | 1 | 5 |
| Relevant part of the image | 104 | 2 | 3,14 | 3,00 | 1,33 | 1,77 | 2 | 4 | 1 | 5 |
| Additional information about office hours or special offers | 101 | 5 | 2,46 | 2,00 | 1,07 | 1,15 | 1 | 4 | 1 | 5 |
| Logos | 96 | 10 | 2,03 | 2,00 | 1,07 | 1,14 | 2 | 4 | 1 | 5 |
| Seals or awards | 91 | 15 | 1,67 | 1,00 | 0,92 | 0,84 | 1 | 4 | 1 | 5 |
| Newsletter elements | 89 | 17 | 1,54 | 1,00 | 0,81 | 0,66 | 1 | 4 | 1 | 5 |

**Table 4: Results block D External Impact of DS. Analysis of interviews with 106 outpatient physicians in Germany (March–October 2018) on qualitative requirements for medical discharge summaries from the recipients’ perspective.**

**Figure 4: Rating distribution block D External Impact of DS. Analysis of interviews with 106 outpatient physicians in Germany (March–October 2018) on qualitative requirements for medical discharge summaries from the recipients’ perspective.**

| **Item** | **n total** | **n missing** | **Mean** | **Median** | **SD** | **Variance** | **Interquartile Range** | **Range** | **Minimum** | **Maximum** |
| --- | --- | --- | --- | --- | --- | --- | --- | --- | --- | --- |
| Logical and coherent line of reasoning | 106 | 0 | 4,49 | 5,00 | 0,74 | 0,55 | 1 | 3 | 2 | 5 |
| Meaningful and clear structure | 106 | 0 | 4,24 | 4,00 | 0,83 | 0,69 | 1 | 3 | 2 | 5 |
| Presentation that makes recipients feel like colleagues | 105 | 1 | 3,74 | 4,00 | 1,05 | 1,11 | 2 | 4 | 1 | 5 |
| Correct German spelling | 106 | 0 | 3,64 | 4,00 | 0,95 | 0,91 | 1 | 3 | 2 | 5 |
| Proper grammar | 106 | 0 | 3,61 | 4,00 | 1,02 | 1,05 | 1 | 4 | 1 | 5 |
| Use of medical terminology | 103 | 3 | 3,42 | 3,00 | 0,96 | 0,93 | 1 | 4 | 1 | 5 |
| Comprehensibility for patients | 104 | 2 | 2,58 | 3,00 | 0,99 | 0,98 | 1 | 4 | 1 | 5 |

**Table 5: Results block E Structure and Linguistic Form. Analysis of interviews with 106 outpatient physicians in Germany (March–October 2018) on qualitative requirements for medical discharge summaries from the recipients’ perspective.**

**Figure 5: Rating distribution block E Structure and Linguistic Form. Analysis of interviews with 106 outpatient physicians in Germany (March–October 2018) on qualitative requirements for medical discharge summaries from the recipients’ perspective.**

| **Item** | **n total** | **n missing** | **Mean** | **Median** | **SD** | **Variance** | **Interquartile Range** | **Range** | **Minimum** | **Maximum** |
| --- | --- | --- | --- | --- | --- | --- | --- | --- | --- | --- |
| Timely or prompt arrival of DS | 106 | 0 | 4,58 | 5,00 | 0,68 | 0,47 | 1 | 4 | 1 | 5 |
| Warnings, e.g., regarding self-harm or harm to others | 106 | 0 | 4,32 | 5,00 | 0,86 | 0,75 | 1 | 4 | 1 | 5 |
| Final DS if a preliminary has been sent | 106 | 0 | 4,24 | 5,00 | 1,03 | 1,07 | 1 | 4 | 1 | 5 |
| Markings of changes in the final DS | 106 | 0 | 3,99 | 4,00 | 1,02 | 1,05 | 2 | 4 | 1 | 5 |
| Patient's preferences regarding treatment proposals | 106 | 0 | 3,40 | 4,00 | 1,04 | 1,09 | 1 | 4 | 1 | 5 |
| Information provided to a patient and his family | 106 | 0 | 3,10 | 3,00 | 1,02 | 1,04 | 1 | 4 | 1 | 5 |
| Formatting and layout | 103 | 3 | 3,01 | 3,00 | 1,04 | 1,08 | 2 | 4 | 1 | 5 |
| Psychological and emotional reactions of patients to an inpatient stay | 104 | 2 | 2,74 | 3,00 | 0,96 | 0,93 | 1 | 4 | 1 | 5 |

**Table 6: Results block F Miscellaneous. Analysis of interviews with 106 outpatient physicians in Germany (March–October 2018) on qualitative requirements for medical discharge summaries from the recipients’ perspective.**

**Figure 6: Rating distribution block F Miscellaneous. Analysis of interviews with 106 outpatient physicians in Germany (March–October 2018) on qualitative requirements for medical discharge summaries from the recipients’ perspective.**

| **Group** | **All physicians** | **Primary care physicians** | **Specialists** |
| --- | --- | --- | --- |
| **Color** |  |  |  |

**Figure 7: Ranking of items in group comparison. Analysis of interviews with 106 outpatient physicians in Germany (March–October 2018) on qualitative requirements for medical discharge summaries from the recipients’ perspective.**


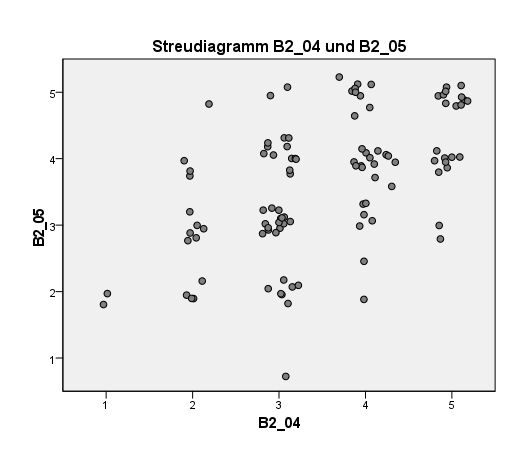


**Figure 8: Scatter plot of the correlation of items B2_04 (Course of a hospital stay) and B2_05 (Explanation for symptoms leading to admission), correlation coefficient 0.561. Analysis of interviews with 106 outpatient physicians in Germany (March–October 2018) on qualitative requirements for medical discharge summaries from the recipients’ perspective.**

| **Item** | **Mean** | **Median** | **Standard deviation** | **Interquartile range** | **Minimum** | **Maximum** | **Range** |
| --- | --- | --- | --- | --- | --- | --- | --- |
| Course of a hospital stay | 3,51 | 3 | 1,01 | 1 | 1 | 5 | 4 |
| Explanation for symptoms leading to admission | 3,64 | 4 | 1,00 | 1 | 1 | 5 | 4 |

**Table 7: Descriptive data on the correlating items B2_04 (Course of a hospital stay) and B2_05 (Explanation for symptoms leading to admission). Analysis of interviews with 106 outpatient physicians in Germany (March–October 2018) on qualitative requirements for medical discharge summaries from the recipients’ perspective.**


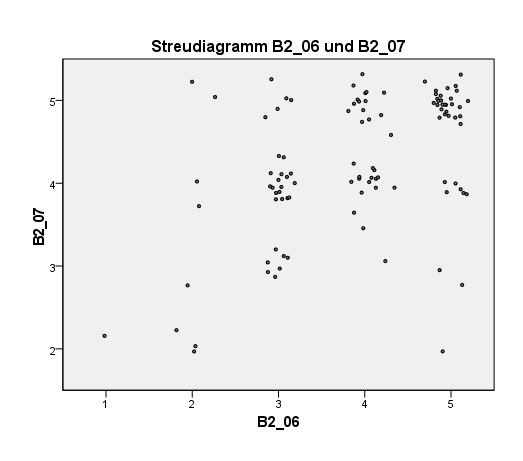


**Figure 9: Scatter plot of the correlation if items B2_06 (Reasons for prescribed medication) and B2_07 (Changes in medication compared to admission), correlation coefficient 0.512. Analysis of interviews with 106 outpatient physicians in Germany (March–October 2018) on qualitative requirements for medical discharge summaries from the recipients’ perspective.**

| **Item** | **Mean** | **Median** | **Standard deviation** | **Interquartile range** | **Minimum** | **Maximum** | **Range** |
| --- | --- | --- | --- | --- | --- | --- | --- |
| Reasons for prescribed medication | 3,9 | 4 | 1,00 | 2 | 1 | 5 | 4 |
| Changes in medication compared to admission | 4,27 | 4 | 0,83 | 1 | 2 | 5 | 3 |

**Table 8: Descriptive data on the correlating items B2_06 (Reasons for prescribed medication) and B2_07 (Changes in medication compared to admission). Analysis of interviews with 106 outpatient physicians in Germany (March–October 2018) on qualitative requirements for medical discharge summaries from the recipients’ perspective.**


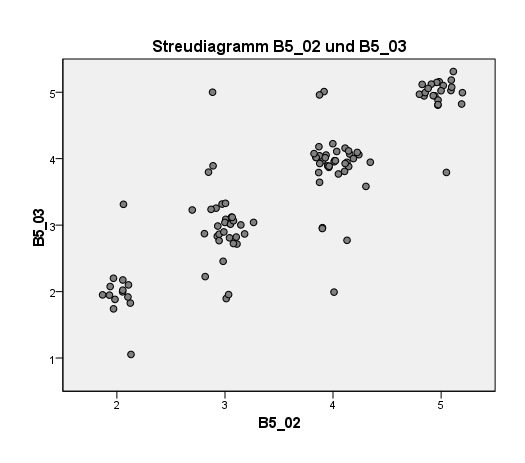


**Figure 10: Scatter plot of the correlation if items B5_02 (Correct German spelling) and B5_03 (Proper grammar), correlation coefficient 0.901. Analysis of interviews with 106 outpatient physicians in Germany (March–October 2018) on qualitative requirements for medical discharge summaries from the recipients’ perspective.**

| **Item** | **Mean** | **Median** | **Standard deviation** | **Interquartile range** | **Minimum** | **Maximum** | **Range** |
| --- | --- | --- | --- | --- | --- | --- | --- |
| Correct German spelling | 3,64 | 4 | 0,95 | 1 | 2 | 5 | 3 |
| Proper grammar | 3,61 | 4 | 1,02 | 1 | 1 | 5 | 4 |

**Table 9: Descriptive data on the correlating items B5_02 (Correct German spelling) and B5_03 (Proper grammar). Analysis of interviews with 106 outpatient physicians in Germany (March–October 2018) on qualitative requirements for medical discharge summaries from the recipients’ perspective.**


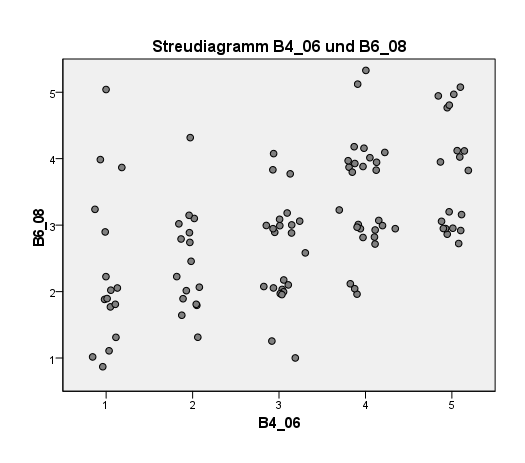


**Figure 11: Scatter plot of the correlation if items B4_06 (Relevant part of the image) and B6_08 (Formatting and layout), correlation coefficient 0.520. Analysis of interviews with 106 outpatient physicians in Germany (March–October 2018) on qualitative requirements for medical discharge summaries from the recipients’ perspective.**

| **Item** | **Mean** | **Median** | **Standard deviation** | **Interquartile range** | **Minimum** | **Maximum** | **Range** |
| --- | --- | --- | --- | --- | --- | --- | --- |
| Relevant part of the image | 3,14 | 3 | 1,33 | 2 | 1 | 5 | 4 |
| Formatting and layout | 3,01 | 3 | 1,04 | 2 | 1 | 5 | 4 |

**Table 10: Descriptive data on the correlating items B4_06 (Relevant part of the image) and B6_08 (Formatting and layout). Analysis of interviews with 106 outpatient physicians in Germany (March–October 2018) on qualitative requirements for medical discharge summaries from the recipients’ perspective.**


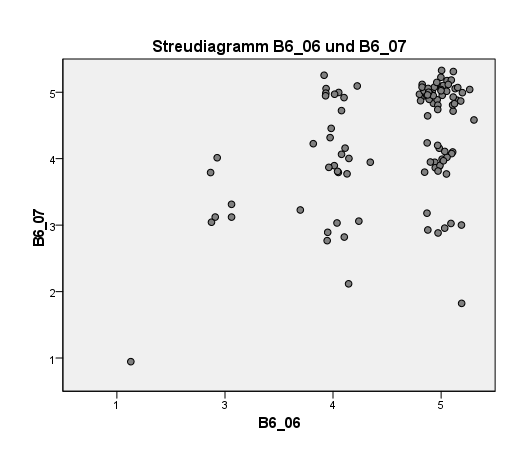


**Figure 12: Scatter plot of the correlation if items B6_06 (Timely or prompt arrival of DS) and B6_07 (Warnings, e.g., regarding self-harm or harm to others), correlation coefficient 0.512. Analysis of interviews with 106 outpatient physicians in Germany (March–October 2018) on qualitative requirements for medical discharge summaries from the recipients’ perspective.**

| **Item** | **Mean** | **Median** | **Standard deviation** | **Interquartile range** | **Minimum** | **Maximum** | **Range** |
| --- | --- | --- | --- | --- | --- | --- | --- |
| Timely or prompt arrival of DS | 4,58 | 5 | 0,68 | 1 | 1 | 5 | 4 |
| Warnings, e.g., regarding self-harm or harm to others | 4,32 | 5 | 0,86 | 1 | 1 | 5 | 4 |

**Table 11: Descriptive data on the correlating items B6_06 (Timely or prompt arrival of DS) and B6_07 (Warnings, e.g., regarding self-harm or harm to others). Analysis of interviews with 106 outpatient physicians in Germany (March–October 2018) on qualitative requirements for medical discharge summaries from the recipients’ perspective.**
